# Investigation of porcine circovirus type 2 and porcine circovirus type 3 infections based on dual TaqMan fluorescent quantitative PCR method and genetic evolutionary analysis of these two viruses

**DOI:** 10.3389/fmicb.2024.1385137

**Published:** 2024-03-14

**Authors:** Mengxiang Cao, Yanwu Wei, Weilin Shi, Li Feng, Liping Huang

**Affiliations:** ^1^Division of Swine Digestive System Infectious Diseases, State Key Laboratory for Animal Disease Control and Prevention, Harbin Veterinary Research Institute, Chinese Academy of Agricultural Sciences, Harbin, China; ^2^Harbin HARVAC Biotechnology Co., Ltd., Harbin, China

**Keywords:** porcine circovirus type 2, porcine circovirus type 3, dual TaqMan fluorescent quantitative PCR, genetic evolution, healthy pig, diseased pig

## Abstract

**Introduction:**

Porcine circovirus type 2 (PCV2) is the pathogen of Porcine Circovirus Associated Diseases. Porcine circovirus type 3 (PCV3) is a novel porcine circovirus associated with porcine dermatitis and nephropathy syndrome (PDNS) and reproductive failure. PCV2 is clearly pathogenic, while the pathogenicity of PCV3 remains controversial, so it is crucial to monitor the prevalence of PCV2 and PCV3 in healthy and diseased pigs to investigate the effects of PCV3 and PCV2 on the health status of pigs.

**Methods:**

Here, we developed a PCV2 and PCV3 dual TaqMan quantitative PCR (qPCR) method to test samples from healthy and diseased pigs, to clarify the differences in the positive rates and viral copy numbers of PCV2 and PCV3, and to analyze the genetic evolution and molecular characterization of the viral genomes obtained with sequence alignment and phylogenetic analysis, homology and structural analysis of Cap proteins, and selection pressure analysis.

**Results:**

We successfully established a dual TaqMan qPCR method for PCV2 and PCV3 with good repeatability, specificity and sensitivity. In total, 1,385 samples from 15 Chinese provinces were tested with the established qPCR. The total positive rates were 37.47% for PCV3 and 57.95% for PCV2, and the coinfection rate for was 25.49%. The positive rates of PCV3 and PCV2 in 372 healthy pigs were 15.05 and 69.89%, respectively, and the coinfection rate was 12.90%. The positive rates of PCV3 and PCV2 in 246 diseased pigs were 55.69 and 83.33%, respectively, and the coinfection rate was 47.97%. Eighteen PCV3 genomes and 64 PCV2 genomes were identified, including nine each of the PCV3a-1 and PCV3b genotypes, eight of PCV2a, 16 of PCV2b, and 40 of PCV2d. The amino acid identity within the PCV3 Cap proteins was 94.00–100.0%, whereas the PCV2 Cap proteins showed an identity of 81.30–100.0%. PCV3 Cap was most variable at amino acid sites 24, 27, 77, 104 and 150, whereas PCV2 Cap had 10–13 unique sites of variation between genotypes.

**Discussion:**

These results clarify the prevalence and variations of PCV2 and PCV3 in healthy and diseased pigs, which will provide a basis for the prevention and control of the two viral infections.

## 1 Introduction

Porcine circovirus (PCV), a member of the genus *Circovirus* in the family *Circoviridae*, is a covalently closed single-stranded circular DNA virus with a particle diameter of approximately 17 nm (Tischer et al., 1982). Four PCVs have been reported: porcine circovirus type 1 (PCV1), porcine circovirus type 2 (PCV2), porcine circovirus type 3 (PCV3), and porcine circovirus type 4 (PCV4). PCV1 was initially detected as a contaminant of serially passaged porcine kidney cells, and antibodies to it are prevalent in swine herds around the world. However, studies have shown that PCV1 is not pathogenic in pigs (Tischer et al., 1974, 1986). In the 1990s, researchers isolated a novel pathogenic PCV from pigs suffering postweaning multisystemic wasting syndrome (PMWS), with <80% nucleotide identity and antigenic differences with PCV1, which was named PCV2 (Allan et al., 1998; Hamel et al., 1998; Meehan et al., 1998). PCV2 often forms coinfections with other pathogens, resulting in severe clinical disease (Allan et al., 1999; Allan and Ellis, 2000). PCV3 is a novel PCV discovered during the macrogenomic sequencing of aborted fetuses from sows with dermatitis and nephropathy (Palinski et al., 2017; Chen et al., 2019). PCV4, with a total length of 1,770 nucleotides (nt), was discovered in pigs with severe clinical signs in Hunan Province, China, in 2019, and unlike PCV2 and PCV3, shares a high degree of homology with mink circovirus (Li et al., 2022). PCV4 was rescued with infectious cloning and its pathogenicity was studied in pigs. Although the challenged piglets showed no obvious clinical signs, significant pathological changes were observed in their organs and PCV4 was detected in almost all tissues (Niu et al., 2022).

The PCV2 genome is 1,766–1,777 nt in length and includes 11 open reading frames (ORFs), of which ORF1 and ORF2 have been functionally defined. ORF1 encodes a replicase-associated (Rep) protein and ORF2 encodes a unique viral capsid (Cap) protein. According to the phylogenetic definition of genotypes proposed by Franzo et al., PCV2 is divided into eight genotypes, PCV2a–PCV2h (Olvera et al., 2007; Sola et al., 2018). The PCV3 genome is 2,000 nt in length and contains three ORFs. ORF1 encodes a Rep protein essential for viral replication, and ORF2 encodes the only structural protein of the virus, the Cap protein. The start codon of ORF3 is unclear, and the function of the encoded protein is unknown. According to the genotyping method of Li et al. (2018), PCV3 is divided into two branches, PCV3a and PCV3b. The PCV3a branch is divided into three sub-branches, PCV3a-1, PCV3a-2, and an IM branch. PCV3 shares <50% genomic identity with PCV2, and they share 26-36% amino acid identity in the Cap protein and 48% in the Rep protein (Rosario et al., 2017; Tan et al., 2021).

PCV2 is pathogenic and the syndromes it causes are mainly postweaning multisystemic wasting syndrome (PMWS), porcine dermatitis and nephropathy syndrome (PDNS), respiratory syndrome, reproductive disorders, and proliferative enteritis (Opriessnig et al., 2007). However, the pathogenicity of PCV3 is controversial due to the difficulty of isolating and culturing the virus. Several studies have shown that it can cause PDNS (Jiang et al., 2019), reproductive disorders, cardiac and multi-system inflammation (Phan et al., 2016), congenital tremors in newborn piglets (Chen et al., 2017), and porcine respiratory disease complex (PRDC) (Kedkovid et al., 2018). In contrast, some studies have shown that it is not pathogenic (Wen et al., 2018; Zheng et al., 2018; Mora-Díaz et al., 2020; Temeeyasen et al., 2020). Although the pathogenicity of PCV3 is controversial, epidemiological studies have shown that it occurs widely in swine herds around the world, with positive rates of 7.41–70.00% in healthy herds (Chen et al., 2022). Coinfection with PCV3 and PCV2 has also been observed, with prevalence rates of 3.4–11.0% (Vargas-Bermudez et al., 2022; Yang et al., 2022). Not many studies have been conducted on the comparison of PCV2 and PCV3 positive and co-infection rates in healthy and diseased pigs in China.

In this study, a dual TaqMan fluorescent quantitative PCR (qPCR) for the simultaneous detection of PCV2 and PCV3 was established to test serum and organ samples from diseased and healthy pigs. The positive rates, viral DNA copy numbers, and representative sequences were compared and analyzed to clarify the prevalence, genetics, and variations in PCV2 and PCV3 in Chinese swine herds. The effects of PCV3 and its coinfection with PCV2 on the health status of swine herds were comprehensively evaluated to provide technical support for the prevention and control of these two pathogens.

## 2 Materials and methods

### 2.1 Samples

A total of 1,385 samples were collected from pig herds in 15 provinces of China: Shaanxi, Heilongjiang, Liaoning, Jilin, Jiangxi, Inner Mongolia, Yunnan, Jiangsu, Hebei, Shandong, Guangdong, Guangxi, Henan, Hunan, and Xinjiang. They included 767 serum samples and 618 organ samples (246 from diseased pigs and 372 from healthy finishers in slaughterhouses). PCV2d-LNHC (GenBank: OL452027), porcine circovirus type 1 (PCV1), porcine pseudorabies virus (PRV), African swine fever virus (ASFV), classical swine fever virus (CSFV), porcine reproductive and respiratory syndrome virus (PRRSV), porcine epidemic diarrhea virus (PEDV), porcine parvovirus (PPV), *Mycoplasma hyopneumoniae* (Mhp), and *Mycoplasma hyorhinis* (Mhr) were isolated, identified, stored at the Harbin Veterinary Research Institute (HVRI) of the Chinese Academy of Agricultural Sciences (CAAS) in Heilongjiang Province, China, and used to assess the specificity of the dual fluorescent qPCR.

### 2.2 Design and synthesis of primers and probes

Specific primers and TaqMan probes were designed according to the conserved regions of the GenBank-registered gene sequences of PCV2 (HM038034, KX828581, and AF055394) and PCV3 (KT869077, MN431644, and KY996341) reference strains (Table 1). The primers and probes were synthesized by GeneScript Biotech Corporation (Nanjing, China).

**Table 1:** Primers and probes were used in this study.

Primer/Probe	Sequence (5′–3′)	Product size (bp)
PCV2-QF169	GTCGACTGTTCTGTAGC	257
PCV2-QR394	CATGGTGAAGAAGTGGTTG	
PCV2-Probe	FAM-AGCAAATGGGCTGCTAATTTTGCAGAC-TAMRA	
QPCV3-F	ACAGCCGTTACTTCACCCCCAAACC	187
QPCV3-R	GTGCCGTAGAAGTCTGTCATTCCAG	
PCV3-Probe	VIC-ATTGAACGGTGGGGTCATATGTGTTGAGCCA-BHQ-1	
PCV2-CF	GGAAGCTTCAGTAATTTATTTCATATGGAA	1,766–1,768
PCV2-CR	GGAAGCTTTTTTATCACTTCGTAATGGTT	
PCV3-Cap-F	CGCGGATCCATGAGACACAGAGCTATATTCAGAA	645
PCV3-Cap-R	CCCAAGCTTTTAGAGAACGGACTTGTAACGAATC	
PCV3-F958	GGACTAGTAATGTTGTACCGGAGGAG	2,000
PCV3-F964	GGACTAGTAATATATAATACCTTAGCCACAAAATTAAC	

### 2.3 Viral nucleic acid extraction and preparation of recombinant plasmid standards

The DNA of PCV3-positive samples, strain PCV2-LNHC, PRV, ASFV, PPV, Mhp, and Mhr cultures were extracted with the TIANamp Genomic DNA Kit (Tiangen, Beijing, China) and the RNA of CSFV, PRRSV, and PEDV cultures were extracted with the Simply P Total Extraction Kit (Bioflux, Hangzhou, China). The RNA of CSFV, PRRSV, and PEDV were reverse transcribed to cDNA with the PrimeScript RT Master Mix (Takara, Dalian, China), and stored at −20°C, together with the other pathogen DNAs. The DNA extracted from strain PCV2-LNHC and PCV3-positive material were used as templates. The target fragments of the corresponding viruses were amplified with PCR using PCV2 primers PCV2-CF/PCV2-CR and PCV3 primers PCV3-F958/PCV3-R964 and then cloned into the pMD18T vector (Takara, Dalian, China) to construct recombinant plasmids containing PCV2 or PCV3 genome. After they were identified with enzyme digestion and DNA sequencing, the plasmids were designated pMD18T-PCV2 and pMD18T-PCV3, respectively, and were used as plasmid standards. The concentrations of the two recombinant plasmids were determined with ultraviolet (UV) spectrophotometry (ng/μL) and converted to copy numbers (copies/μL) according to the formula: copies/μL = $\frac{\left(6.02\times 10^{23}\right)\times \left(Χ\;\frac{\mathrm{ng}}{\mathrm{\mu L}}\times 10^{-9}\right)}{DNA\;\mathrm{length}\;\left(\mathrm{bp}\right)\times 660}$ .

### 2.4 Establishment of PCV2 and PCV3 dual TaqMan fluorescent qPCR

The PCV2 and PCV3 standards at final concentrations of 10^3^ copies/μL and 10^4^ copies/μL, respectively, were mixed in equal volumes as the PCR templates, and the dual fluorescent qPCR was performed under the same conditions using two pairs of primers and the corresponding TaqMan probes. A checkerboard titration method was used to determine the amplification effects of different concentrations of primers (1, 0.8, 0.6, 0.4, or 0.2 μmol/L), probes (1, 0.8, 0.6, 0.4, or 0.2 μmol/L), and annealing temperatures (56, 57, 58, 59, or 60°C) to determine the optimal reaction conditions for the dual fluorescent qPCR.

The PCV2 and PCV3 plasmid standards at different concentrations (1 × 10^9^, 1 × 10^8^, 1 × 10^7^, 1 × 10^6^, 1 × 10^5^, 1 × 10^4^, or 1 × 10^3^ copies/μL) were selected as templates, and the optimized reaction conditions were used for the dual fluorescent qPCR assay, with three replicates of each dilution, to construct standard curves of PCV2 and PCV3 with the dual fluorescent qPCR.

### 2.5 Validation of PCV2 and PCV3 dual TaqMan fluorescent qPCR

To evaluate the specificity of this dual qPCR, DNAs or cDNAs extracted from PCV1, PRV, ASFV, CSFV, PRRSV, PEDV, PPV, Mhp, and Mhr cultures were amplified under the optimal conditions. The nucleic acids of PCV2 and PCV3 were used as the positive controls, and double-distilled H_2_O (ddH_2_O) was used as the negative control.

To evaluate the sensitivity of the established assay, different concentrations of the standard plasmids were tested with the method as follows. The same concentrations of PCV2 and PCV3 standard plasmids were mixed in equal volumes and then diluted to form a 10-fold gradient, and dilutions of 5 × 10^8^–5 × 10^0^ copies/μL of the plasmid standards were selected as templates for the established TaqMan fluorescent qPCR. Three replicates were established for each dilution, and ddH_2_O was used as the negative control.

The PCV2 and PCV3 standard plasmids with final concentrations of 5 × 10^7^, 5 × 10^6^, and 5 × 10^5^ copies/μL were selected as templates to test the intra-batch reproducibility of the established dual fluorescent qPCR. Each sample was tested three times and the mean cycle threshold (Ct) value, standard variance, and coefficient of variation of the three replicates were calculated. The same method was used to test the inter-batch reproducibility of the assay with standards prepared from different batches, and the mean Ct value, standard variance, and coefficient of variation of the two amplifications were calculated.

### 2.6 Testing clinical samples

To determine the PCV2 and PCV3 DNA loads in the pig samples, 1 g of tissue sample was ground in a sterilized mortar to make an emulsion sample by adding 5 mL of Dulbecco’s modified Eagle’s medium (DMEM). Viral DNA was extracted from the emulsion and serum samples with the TIANamp Genomic DNA Kit (Tiangen, Beijing, China). The PCV2 and PCV3 dual TaqMan fluorescent qPCR established in this study was used to test these samples, and the results for the diseased and healthy pig samples were analyzed statistically.

### 2.7 Amplification and sequencing of PCV2 and PCV3 genomes

To obtain genomic information for PCV2 and PCV3, positive samples with Ct <30 were randomly selected from each geographic region or swine farm from the test samples described above, and the whole genomes of PCV2 and PCV3 or PCV3 ORF2 were amplified with KOD DNA polymerase (Toyobo, Tokyo, Japan) and the PCV2 and PCV3 primers (Table 1; PCV2-CF and PCV2-CR, PCV3-F958 and PCV3-F964 or PCV3-Cap-F and PCV3-Cap-R, respectively), as described previously (Xia et al., 2019). The target fragments were recovered with an E.Z.N.A.^®^ Gel Extraction Kit (Omega, Georgia, United States) and cloned into the pMD18-T vector (Takara, Dalian, China). Five positive plasmids were randomly selected and sequenced by Comate Bioscience Co., Ltd. (Changchun, Jilin, China).

### 2.8 Sequence alignment and phylogenetic analysis

Sixty-four newly sequenced whole PCV2 genomes and 25 reference sequences from the GenBank database were used to construct a phylogenetic tree. A multiple-sequence alignment of the nucleotide sequences of complete PCV2 genomes was generated with the ClustalW algorithm in MEGA 7.0. A phylogenetic tree was constructed with the neighbor-joining method with 1,000 bootstraps replicates and the p-distance model, and then modified, annotated and visualized by one table (tvBOT).^1^

For PCV3, a multiple-sequence alignment of the 18 whole PCV3 genomes determined in this study together with 33 reference sequences from the GenBank database was generated with the ClustalW algorithm in MEGA 7.0. Maximum-likelihood (ML) trees were constructed with the Tamura–Nei model with a gamma distribution. A bootstrap analysis with 1,000 replicates was used to determine the reliability of the generated tree. Finally, the tree was also modified, annotated and visualized by one table (tvBOT) (see text footnote 1). A phylogenetic tree based on PCV3 ORF2 was also constructed and modified as described above.

### 2.9 Selection pressure analysis of Rep and Cap

To determine whether selection pressure has led to the variability observed in PCV2 and PCV3, a selection pressure analysis of the Rep and Cap of PCV2 and PCV3 was conducted with the Datamonkey program.^2^ The methods used to estimate positive codon sites included fixed-effects likelihood (FEL), fast unconstrained Bayesian approximation (FUBAR), single likelihood ancestor counting (SLAC), and mixed-effects model of evolution (MEME). The sites under positive selection were confirmed with at least two methods, with *p*-values of <0.05 for both FEL and MEME and *p <* 0.1 for SLAC, and a posterior probability of *>*0.9 for FUBAR.

### 2.10 Homology analysis of PCV2 or PCV3

To determine the homology among different strains of PCV2 or PCV3, we aligned genomes, ORF1s, ORF2s, Rep proteins and Cap proteins of PCV2 or PCV3 with MegAlign software.

### 2.11 Structural analysis of PCV3 Cap

The PCV3 Cap (accession numbers: PP179129) structure was predicted with I-TASSER website^3^ and the partial virus-like particle (VLP) structure of PCV3 was constructed with a PCV2 VLP template (PDB: 6OLA) using homologous modeling with the SWISS-MODEL server.^4^ The structure was visualized and displayed with UCSF Chimera X. Nonconserved amino acid sites detected in the homology analysis of the Cap proteins were localized on the structure of the PCV3 Cap protein.

### 2.12 Statistical analysis

The GraphPad Prism 9.0 software (GraphPad Software, San Diego, CA, United States) was used for all data analyses. The qPCR data were analyzed with one-way repeated-measures analysis of variance (ANOVA), followed by a least significance difference (LSD) test. A value of *p* < 0.05 was considered to indicate a significant difference between groups.

## 3 Results

### 3.1 Development of PCV2 and PCV3 double TaqMan fluorescent qPCR

Plasmid standards pMD18T-PCV2 and pMD18T-PCV3 were successfully constructed by cloning the full-length PCV2 and PCV3 sequences separately into the pMD18-T vector, respectively.

The reaction conditions for the double qPCR were optimized with a checkerboard method. The optimal reaction system was as follows: 10 μL of *Premix Ex Taq* enzyme (Takara, Dalian, China), 0.8 μL each of the upstream and downstream primers (10 μmol/L) for PCV2 and 1.2 μL each of the upstream and downstream primers (10 μmol/L) for PCV3, 0.8 μL of probe (10 μmol/L), 1 μL of sample DNA, and 3.4 μL of ddH_2_O. The optimal amplification program was predenaturation at 95°C for 30 s, followed by 40 cycles of denaturation at 95°C for 4 s, and annealing and extension at 60°C for 32 s.

**Figure 1:**
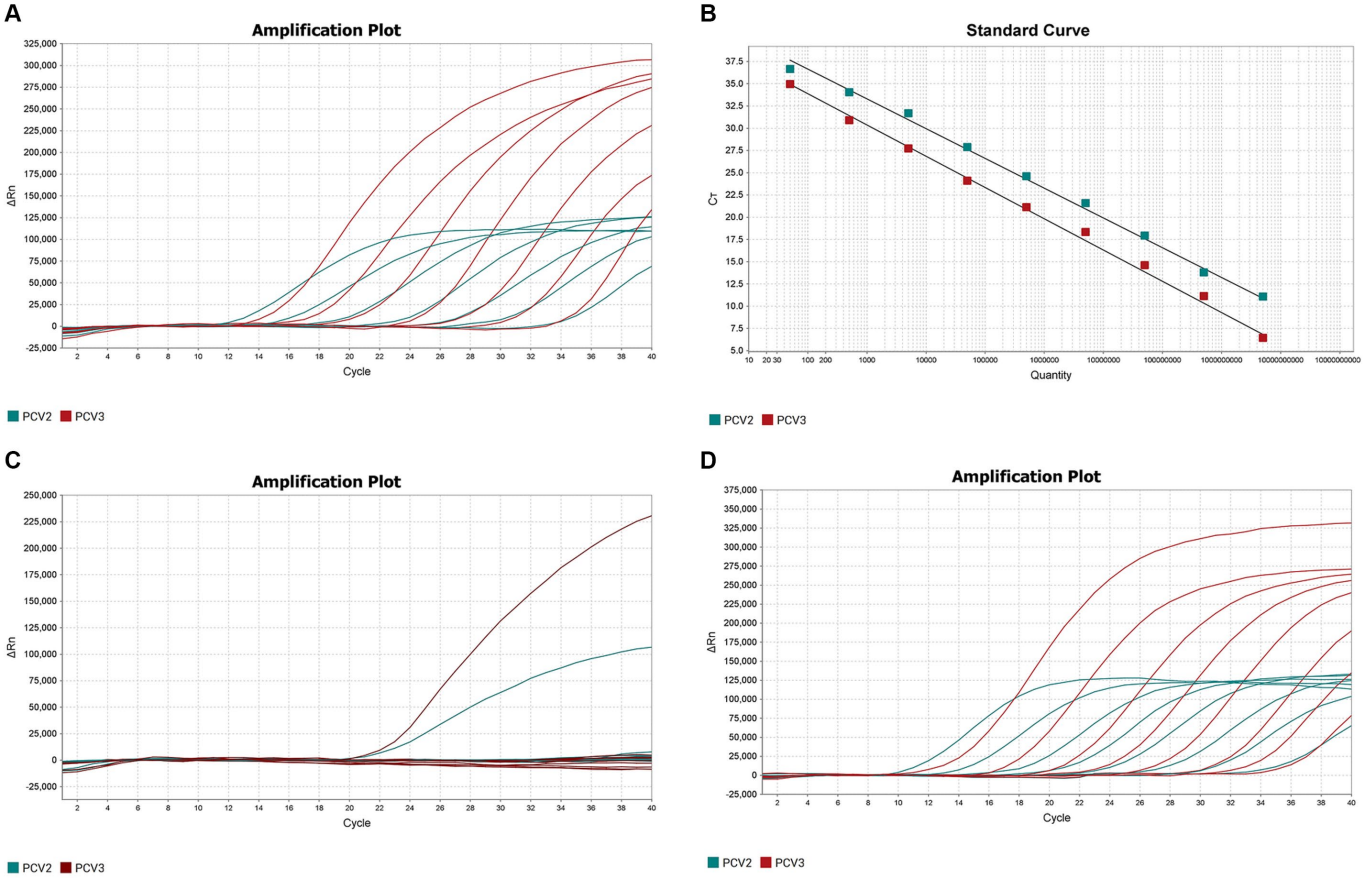
Establishment and validation of PCV2 and PCV3 dual TaqMan fluorescent quantitative PCR. **(A)** Amplification curves for PCV2 and PCV3 at concentrations of 10^9^–10^3^ copies/μL. Red indicates the amplification curve for PCV3, green for PCV2. **(B)** Standard curves for PCV2 and PCV3 were generated under the the optimum amplification conditions. The correlation coefficients and amplification efficacy of the standard curves: PCV2 (slope: −3.346; *y*-intercept: 43.328; *R*^2^: 0.997; amplification efficiency: 99.005%), PCV3 (slope: −3.516; *y*-intercept: 40.922; *R*^2^: 0.996; amplification efficiency: 92.415%) were ideal for detecting the target genes. **(C)** Specificity analysis of PCV2 and PCV3. Two amplification curves represent samples positive for PCV2 and PCV3, and negative samples include PCV1, PRV, ASFV, CSFV, PRRSV, PEDV, PPV, Mhp, Mhr and negative control. **(D)** Sensitivity analysis of PCV2 and PCV3 at concentrations of 5 × 10^8^–5 × 10^0^ copies/μL. The lowest copy number detected with qPCR was 50 copies/μL for both PCV2 and PCV3.

The optimized double fluorescent qPCR was used to amplify the pMD18T-PCV2 and pMD18T-PCV3 standards (templates) at final concentrations of 1 × 10^9^–1 × 10^3^ copies/μL, and the amplification curves (Figure 1A) and standard curves (Figure 1B) were obtained. The amplification efficiencies of PCV2 and PCV3 were 99.005 and 92.415%, respectively, indicating that the amplification efficiencies were high. The correlation coefficients of the standard curves of PCV2 and PCV3 were 0.997 and 0.996, respectively, indicating that the number of copies (1 × 10^9^–1 × 10^3^ copies/μL) of the two standard products showed good linear relationships with the Ct values.

### 3.2 Validation of double TaqMan fluorescent qPCR

The DNA or cDNA of PCV1, PCV2, PCV3, PRV, ASFV, CSFV, PRRSV, PEDV, PPV, Mhp, and Mhr, used as the qPCR templates, were analyzed with the established dual TaqMan fluorescent qPCR. Only PCV2 and PCV3 generated specific amplification curves (Figure 1C), whereas no specific amplification curves were generated for the other pathogens, confirming the specificity of the assay.

The sensitivity of the dual TaqMan fluorescent qPCR was tested with the PCV2 and PCV3 standards at final concentrations of 5 × 10^8^–5 × 10^0^ copies/μL. The results are shown in Figure 1D, 50 copies of PCV2 and PCV3 could be detected simultaneously, with Ct values of 34.96 and 33.99, respectively. However, dilutions containing 5 copies of each target were not detected using the same amplification system. So the lowest limits of detection for PCV2 and PCV3 in the dual fluorescent qPCR were both 50 copies/μL, demonstrating the good sensitivity of the method.

Three concentrations of the mixtures of PCV2 and PCV3 standard plasmids were randomly selected for testing the intra- and inter- batch reproducibility of the dual fluorescent qPCR established in this study (Table 2). The intra-batch coefficient of variation was 0.23–1.20% and the inter-batch coefficient of variation was 0.99–1.94%, confirming the good repeatability of the assay.

**Table 2:** Repeatability of the TaqMan dual fluorescent quantitative PCR.

Pathogens	Concentration (copies/μL)	Intra-batch reproducibility test			Inter-batch reproducibility test		
		Ct means	SD	CV%	Ct means	SD	CV%
PCV3	5 × 10^7^	17.72	0.08	0.46%	17.65	0.18	0.99%
	5 × 10^6^	20.61	0.23	1.14%	20.82	0.24	1.15%
	5 × 10^5^	25.33	0.30	1.20%	25.29	0.34	1.36%
PCV2	5 × 10^7^	20.26	0.04	0.23%	20.21	0.27	1.33%
	5 × 10^6^	23.15	0.18	0.77%	23.57	0.46	1.94%
	5 × 10^5^	26.54	0.31	1.15%	27.28	0.50	1.84%

### 3.3 High detection rates of PCV2 and PCV3 in China

The double TaqMan fluorescent qPCR was used to test 1,385 samples collected from 15 provinces of China: Shaanxi, Heilongjiang, Liaoning, Jilin, Jiangxi, Inner Mongolia, Yunnan, Jiangsu, Hebei, Shandong, Guangdong, Guangxi, Henan, Hunan, and Xinjiang. The results are shown in Table 3. The total positive rate of PCV3 was 37.47%, the total positive rate of PCV2 was 57.95%, and the coinfection rate was 25.49%. The positive rate of PCV3 in the 15 provinces ranged from 6.45 to 76.92% and the positive rate of PCV2 ranged from 0.00 to 83.33%. It can be seen that PCV3 and PCV2 are widely prevalent in China.

**Table 3:** PCV2 and PCV3 positive rates in different provinces.

Provinces	PCV3 positive rate (positive number/sample number)	PCV2 positive rate (positive number/sample number)
Heilongjiang	60.22% (109/181)	62.98% (114/181)
Liaoning	60.81% (45/74)	48.65% (36/74)
Jilin	24.65% (125/507)	43.39% (220/507)
Shanxi	34.29% (36/105)	71.43% (75/105)
Inner Mongolia	40.48% (119/294)	68.71% (202/294)
Shandong	45.21% (33/73)	57.53% (42/73)
Jiangxi	6.45% (4/62)	67.74% (42/62)
Yunnan	20.00% (1/5)	60.00% (3/5)
Hebei	55.56% (10/18)	83.33% (15/18)
Jiangsu	50.00% (3/6)	66.67% (4/6)
Guangxi	50.00% (13/26)	65.38% (17/26)
Guangdong	62.50% (5/8)	62.50% (5/8)
Henan	76.92% (10/13)	61.54% (8/13)
Hunan	57.14% (4/7)	0.00% (0/7)
Xinjiang	33.33% (2/6)	66.67% (4/6)
Total	37.47% (519/1385)	57.95% (787/1385)

### 3.4 Detection rates of PCV2 and PCV3 in different samples

**Figure 2:**
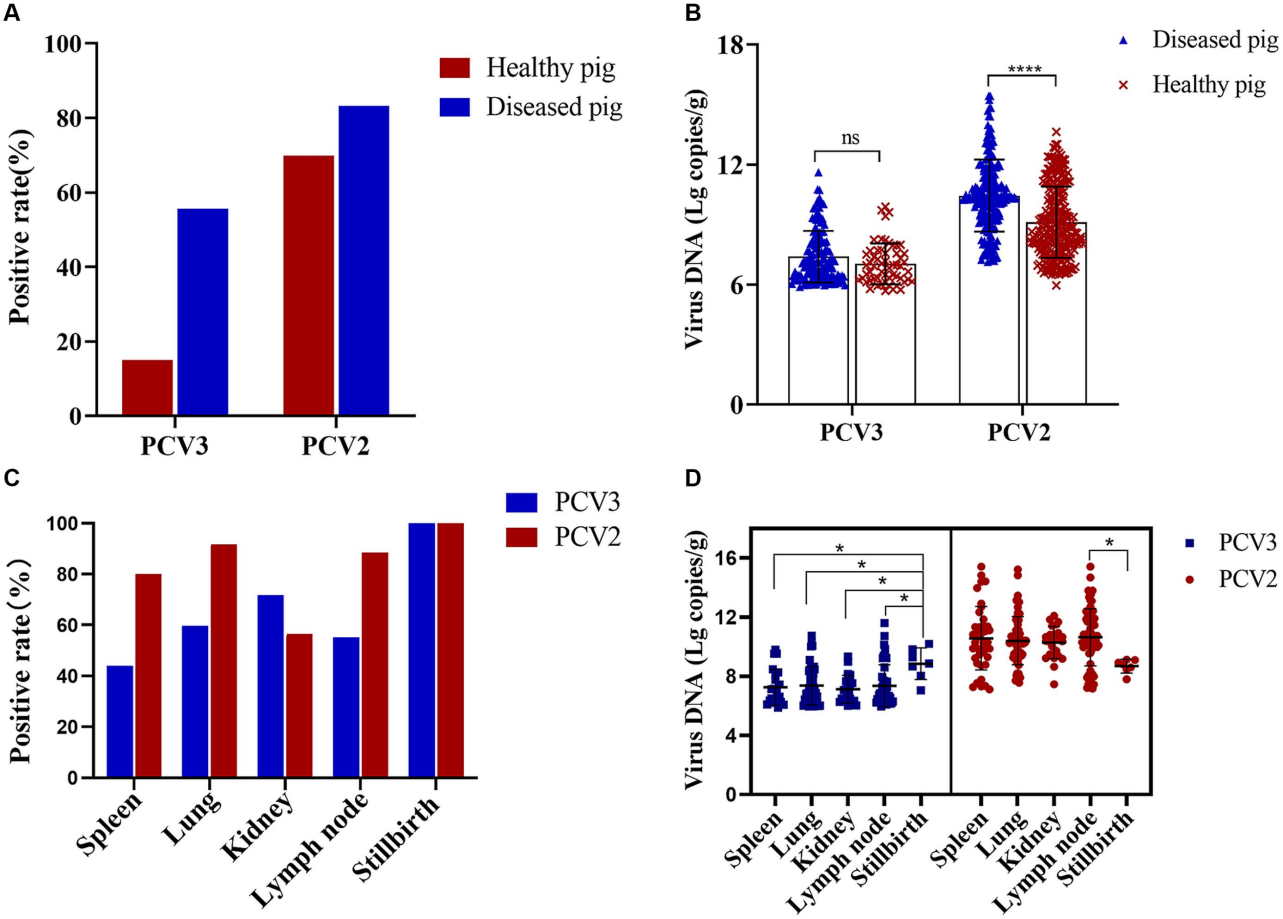
PCV2 and PCV3 positive rates in diseased and healthy pigs and in different organs. **(A)** Positive rates and **(B)** viral loads for PCV2 and PCV3 in diseased and healthy pigs. Red represents positive rates or viral loads in healthy pigs, and blue represents those in diseased pigs. ^****^Indicates statistically significant difference in PCV2 between diseased and healthy pigs (^****^p < 0.0001). **(C)** Positive rates and **(D)** viral loads for PCV2 and PCV3 in different organs. Red represents PCV2 and blue represents PCV3. ^*^Indicates statistically significant difference between different organs (^*^p < 0.05).

A total of 618 organ samples were examined in this study, 372 of which were healthy pig organs (lymph nodes) obtained from slaughterhouses, and 246 of which were various organs from diseased pigs: lymph nodes, lungs, spleens, kidneys, and stillbirths. Fifty-six PCV3-positive and 260 PCV2-positive organs were detected in the 372 healthy pigs, with PCV3 and PCV2 positive rates of 15.05 and 69.89%, respectively, and a coinfection rate of 12.90%. Among the 246 organs from diseased pigs, 137 were positive for PCV3 and 205 were positive for PCV2. The positive rates for PCV3 and PCV2 were 55.69 and 83.33%, respectively, and the coinfection rate was 47.97% (Figure 2A). The mean nucleic acid copy number of PCV2 was significantly higher in the organs of the diseased pigs than in the organs of the healthy pigs, but the nucleic acid copy number of PCV3 did not differ significantly between the healthy pigs and the diseased pigs (Figure 2B).

The positive rates of PCV2 and PCV3 in the different organs of the diseased pigs and their viral loads were also analyzed statistically. PCV3 positive rate was highest in stillbirths, followed by kidneys and lungs, and the viral loads in stillbirths were the highest, which was significantly different from that in other organs (Figures 2C,D). PCV2 was detected in the spleens, lungs, kidneys, lymph nodes, and stillbirths of diseased pigs, and the positive rates were 80.00, 91.67, 56.41, 88.46 and 100.00%, respectively (Figure 2C). The highest viral copy number was found in the lymph nodes, which was significantly different from that in stillbirths, followed by the spleens (Figure 2D).

### 3.5 Coinfections of PCV2 or PCV3 with other porcine viruses

Fifty-six organs from pigs suspected of PCV-associated diseases (PCVAD) in Liaoning, Heilongjiang, Shandong, Jilin, Hebei, Jiangsu, and Inner Mongolia were tested for PCV2, PCV3, CSFV, and PRRSV. The results are shown in Table 4. The coinfection rates of PCV3 with PCV2, PRRSV, or CSFV were 57.14, 35.71%, or 35.90%, respectively, and the coinfection rates of PCV2 with PRRSV or CSFV were 44.64% or 37.50%, respectively. The mixed infection rate with all four viruses was 19.64%. These data demonstrate that mixed infections of PCV2 and PCV3 with other pathogens are common.

**Table 4:** Positive rates of different pathogens.

Pathogens	Number of positive	Positive rates
PCV3/PCV2	32	57.14%
PCV3/CSFV	14	35.90%
PCV3/PRRSV	15	35.71%
PCV3/CSFV/PRRSV	11	23.91%
PCV2/PRRSV	25	44.64%
PCV2/CSFV	21	37.50%
PCV3/PCV2/PRRSV/CSFV	11	19.64%

### 3.6 Phylogenetic analysis of PCV3 and PCV2

A total of 18 full-length PCV3 genomic sequences and 64 full-length PCV2 genomic sequences were obtained and successfully sequenced (accession numbers: PP179064–PP179145).

**Figure 3:**
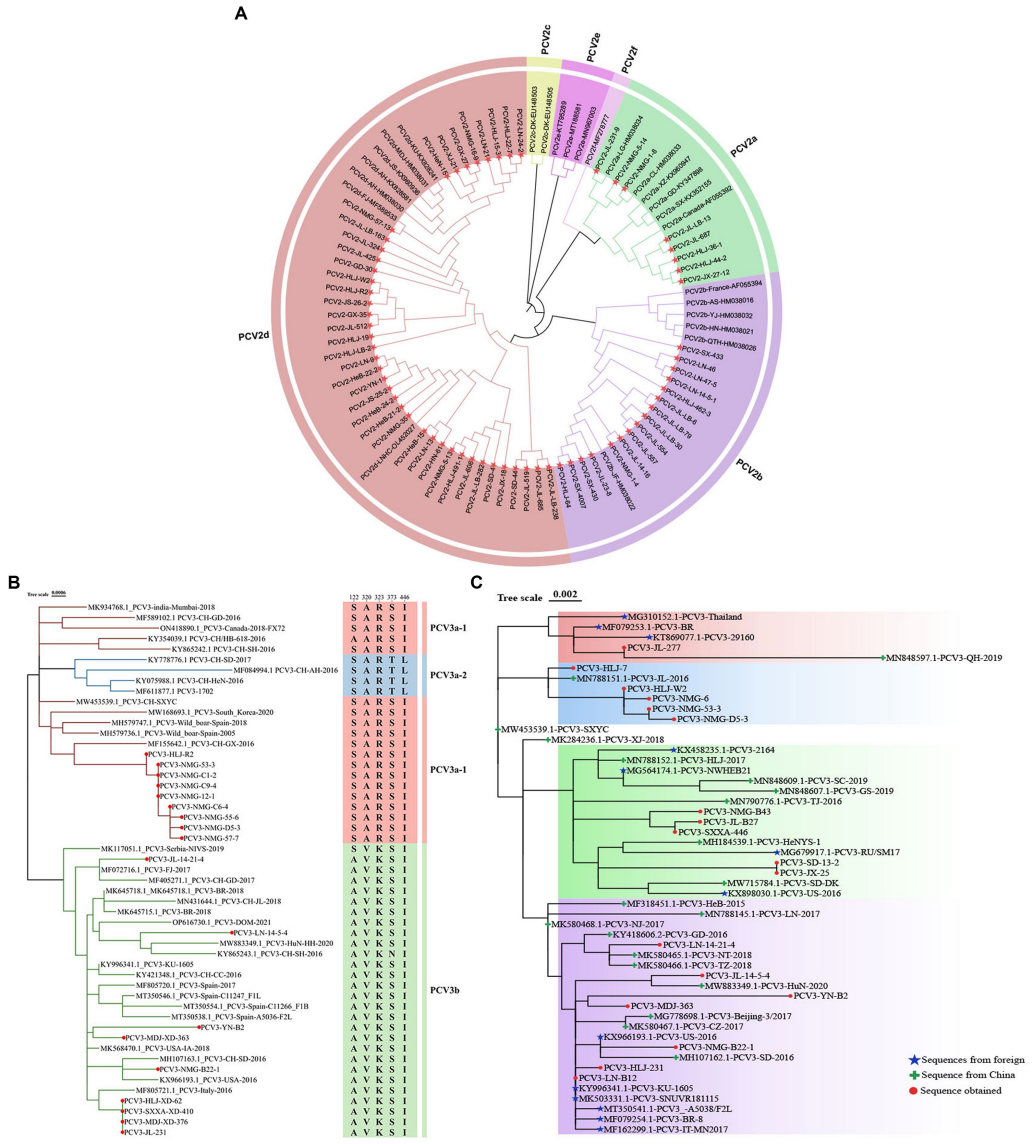
Phylogenetic analysis of PCV2 and PCV3. **(A)** Phylogenetic analysis of 64 complete PCV2 genomes isolated in this study, together with 25 complete PCV2 genomes obtained from GenBank. The tree was constructed with the neighbor-joining method in the MEGA 7.0 software (*p*-distance model; 1,000 bootstrap replicates). Different colors represent different genotypes, and the strains sequenced in this study are marked with red stars. **(B)** Phylogenetic tree based on the complete sequences of 51 PCV3 strains: 18 strains from this study (marked with red circles) and 33 strains from GenBank. The maximum likelihood tree was constructed with the Tamura-Nei model with gamma distribution and 1,000 bootstrap replicates. The PCV3 genotyping was proposed by Li et al. (2018) and all PCV3 strains could be divided into three branches, PCV3a-1 (Red), PCV3a-2 (Blue), and PCV3b (Green). **(C)** Phylogenetic tree based on ORF2 of PCV3. Chinese reference sequences are marked with green crosses; foreign strains are marked with blue stars; and sequences obtained in this study are marked with red circles.

The phylogenetic tree constructed from full-length PCV2 genomes showed that eight of the full-length genomic PCV2 sequences determined in this study were PCV2a, 16 were PCV2b, and 40 were PCV2d, accounting for 12.5, 25.0, and 62.5% of the sequences, respectively (Figure 3A). From this, we inferred that PCV2d is currently the most prevalent PCV2 genotype in the pig populations in these provinces.

The evolutionary tree based on the PCV3 genome showed that nine of the 18 sequences identified were PCV3a-1 and nine were PCV3b (Figure 3B). However, the ORF2 sequences determined in this study were distributed on four different branches of the evolutionary tree based on PCV3 ORF2 (accession numbers: PP179146–PP179163) (Figure 3C). These data suggest that the results of the evolutionary trees drawn based on full length and ORF2 are different. The identity of sequences PCV3-B2, PCV3-231, PCV3-14-21-4, PCV3-B22-1, and PCV3-14-5-4 isolated in the Chinese provinces of Heilongjiang, Yunnan, Liaoning, Inner Mongolia, and Jilin, with the sequences obtained from Guangdong, Jiangsu, Zhejiang, Shandong, and Hunan in China was 98.3–99.7%, and with the sequences originating from South Korea, Spain, Italy, Brazil, and the United States was 97.7–99.8%, respectively (Figure 3C). Thus, the identity of PCV3 sequences originating from different regions of the same country and from different countries was >97.7%, indicating that PCV3 is more conserved than PCV2, and does not differ across geographic regions.

### 3.7 Homology analysis of PCV3 or PCV2

The homology among the sequences determined in this study and the reference sequences was analyzed with the MegAlign software. The nucleotide identity within the PCV3 and PCV2 genomes was 98.4–99.8% and 94.5–100%, respectively. The sequence identity of ORF1 and its encoded proteins (Rep) within PCV3 was 98.1–100% and 96.6–100%, respectively, whereas that of ORF1 and its encoded proteins of PCV2 shared 97.0–100% and 98.1–100% sequence identity, respectively. The sequence identity of 96.3–100% and 94.0–100% were observed for ORF2 and its encoded proteins (Cap) within PCV3, whereas 86.4–100% and 81.3–100% sequence identity were observed for ORF2 and the encoded proteins within PCV2, respectively. Therefore, the mutation rate of PCV2 is higher than that of PCV3, and the mutation rate of ORF2 is higher than that of ORF1 in both PCV2 and PCV3.

### 3.8 Analysis of PCV2 and PCV3 Rep proteins

**Figure 4:**
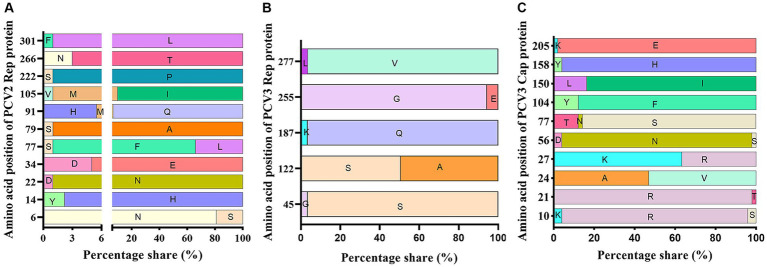
Amino acid analysis of PCV3 Rep and Cap proteins and PCV2 Rep protein. Rep and Cap proteins derived from 51 PCV3 genomes and Rep proteins derived from 89 PCV2 genomes were aligned with ClustalX-2.1. The variable sites and the probabilities of variable amino acids were counted and presented with GraphPad 8.0. **(A)** PCV2 Rep protein. **(B)** PCV3 Rep protein. **(C)** PCV3 Cap protein.

The amino acid sequences of the Rep proteins of PCV2 and PCV3 were aligned using ClustalX-2.1. The results showed 11 and 5 variable sites in the PCV2 and PCV3 Rep proteins, respectively (Figures 4A,B). PCV3 Rep had a higher likelihood of mutation at amino acids 122 and 255, whereas PCV2-Rep protein had more frequent mutations at amino acids 6, 77, 91 and 105 (Figures 4A,B).

### 3.9 Analysis of PCV2 and PCV3 Cap proteins

The amino acid sequences of the PCV3 and PCV2 Cap proteins were analyzed with ClustalX-2.1. Ten amino acid variable sites were detected in PCV3 Cap, and the most frequently mutated sites were at positions 24, 27, 77, 104 and 150 (Figure 4C). Unlike PCV3 Cap, which is highly conserved, PCV2 Cap had 37 variable sites, most of these sites were located in the loop region of the Cap protein and 56.76% (21/37) were exposed on the surface of the VLP, with most-frequent changes at amino acid positions 53, 57, 59, 68, 88, 89, 90, 91, 121, 151, 169, 190, 210, 215, 217, 232, and 234 (Table 5). Among these, 10 unique amino acid sites varied between PCV2a and PCV2b: ^57^V → I, ^59^A → R/K, ^63^S/R/T → R/K, ^88^K → P, ^89^I → R, ^91^I → R, ^151^P → T, ^190^S → T/A, ^210^D → E, and ^232^K → N. PCV2a and PCV2d differed at 13 unique amino acid sites: ^53^F → I, ^59^A → K, ^63^S/R/T → R/K, ^68^S/A → N, ^88^K → P, ^89^I → L, ^90^S → T, ^91^I → V, ^151^P → T, ^169^S → R/G, ^190^S → T, ^215^V → I, and ^234^* → K/M. The 11 unique amino acid sites that differed between PCV2b and PCV2d were ^53^F → I, ^57^I → V, ^68^A → N, ^89^R → L, ^90^S → T, ^121^S → T, ^134^T → N, ^169^S → R/G, ^210^E → D, ^215^V → I, and ^234^* → K/M. Although the amino acid identity varied widely between different PCV2 Cap proteins, with a minimum identity of only 81.3%, the PCV2a, PCV2b, and PCV2d Cap proteins showed greater internal identity, in the ranges 90.6–100%, 97.4–100%, and 97.0–100%, respectively. These results indicate that there are significant differences between the Cap proteins of different PCV2 genotypes, but few differences within the genotypes.

**Table 5:** Information on the amino acid variability in PCV2 ORF2.

Amino acid position	PCV2a	PCV2b	PCV2d	^a^Positions in the 3D structure of capsid protein	^b^Outside or inside of the VLP
8	F/Y	Y	F/Y	NLS	Inside
21	Q/L	Q	Q	NLS	Inside
47	A/S/T	T	T	B β-strand	Inside
51	R/S/C	R	R	B β-strand	Outside
53	F	F	I	B β-strand	Outside
57	V	I	V	B β-strand	Outside
59	A	K/R	K	BC-loop	Outside
63	S/R/T	R/K	R/K	BC-loop	Outside
68	S/A	A	N	C β-strand	Outside
72	L/M	M	M	C β-strand	Outside
76	L/I	I	I	α-helice	Inside
77	N/D	N	N	α-helice	Outside
80	V/L	L	L	CD-loop	Inside
86	T/S	S	S	CD-loop	Outside
88	K	P	P	CD-loop	Outside
89	I	R	L	CD-loop	Inside
90	S	S	T	CD-loop	Inside
91	I	V	V	CD-loop	Inside
121	S/T	S	T	E β-strand	Inside
123	V/I	V	V	E β-strand	Inside
131	T/M/P	T	T	EF-loop	Outside
133	V/A	A	A	EF-loop	Outside
134	T/P/N	T	N	EF-loop	Outside
137	Q/L	L	L	EF-loop	Outside
151	P	T	T	F β-strand	Inside
169	S	S	R/G	GH-loop	Outside
185	M/L	L	L	GH-loop	Inside
187	I/L	L	L	GH-loop	Inside
190	S	T/A	T	GH-loop	Outside
191	A/R/G	G	G	GH-loop	Outside
200	T/I	T	T	H β-strand	Inside
206	K/I/T	I	I	HI-loop	Outside
210	D	E	D	I β-strand	Outside
215	V	V	I	I β-strand	Inside
217	M	M	M/L	I β-strand	Inside
232	K	N	N/K	C tail	Outside
234	*	*	K/M	C tail	Outside

*: no amino acid at this position.

a Positions of variable amino acids in the 3D structure of capsid protein were listed according to the reference of Khayat et al. (2011).

b Whether the mutant amino acid is located inside or outside the PCV2 VLP was analyzed by UCSF Chimera X with a PCV2 VLP template (PDB: 6OLA).

### 3.10 Selection pressure analysis of PCV3 and PCV2

The ORF1 and ORF2 sequences of PCV3 and PCV2 were analyzed to identify loci subject to selection pressure. As shown in Table 6, amino acid position 91 in PCV2 Rep, positions 63 and 191 in PCV2 Cap, position 122 in PCV3 Rep, and positions 5, 24, 137, and 156 in PCV3 Cap are under positive selection suggesting that they play positive roles in adaptive evolution.

**Table 6:** Selection pressure analysis of Rep and Cap of PCV2 and PCV3.

Coden		*p*-value			Posterior probability
		MEME	FEL	SLAC	FUBAR
PCV3 ORF1	122	**0.000**	**0.021**	0.056	**0.995**
PCV3 ORF2	5	**0.030**	**0.015**	0.080	**0.937**
	24	**0.030**	**0.032**	**0.020**	**0.997**
	137	**0.000**	**0.007**	**0.016**	**0.995**
	156	**0.000**	**0.015**	0.197	**0.955**
PCV2 ORF1	91	**0.050**	0.224	0.335	**0.953**
PCV2 ORF2	63	**0.040**	**0.026**	0.065	**0.988**
	191	**0.050**	**0.047**	0.126	**0.959**

The bold representing the *p* < 0.05 and posterior probability >0.9, with significant difference.

### 3.11 Structural analysis of PCV3 Cap protein

**Figure 5:**
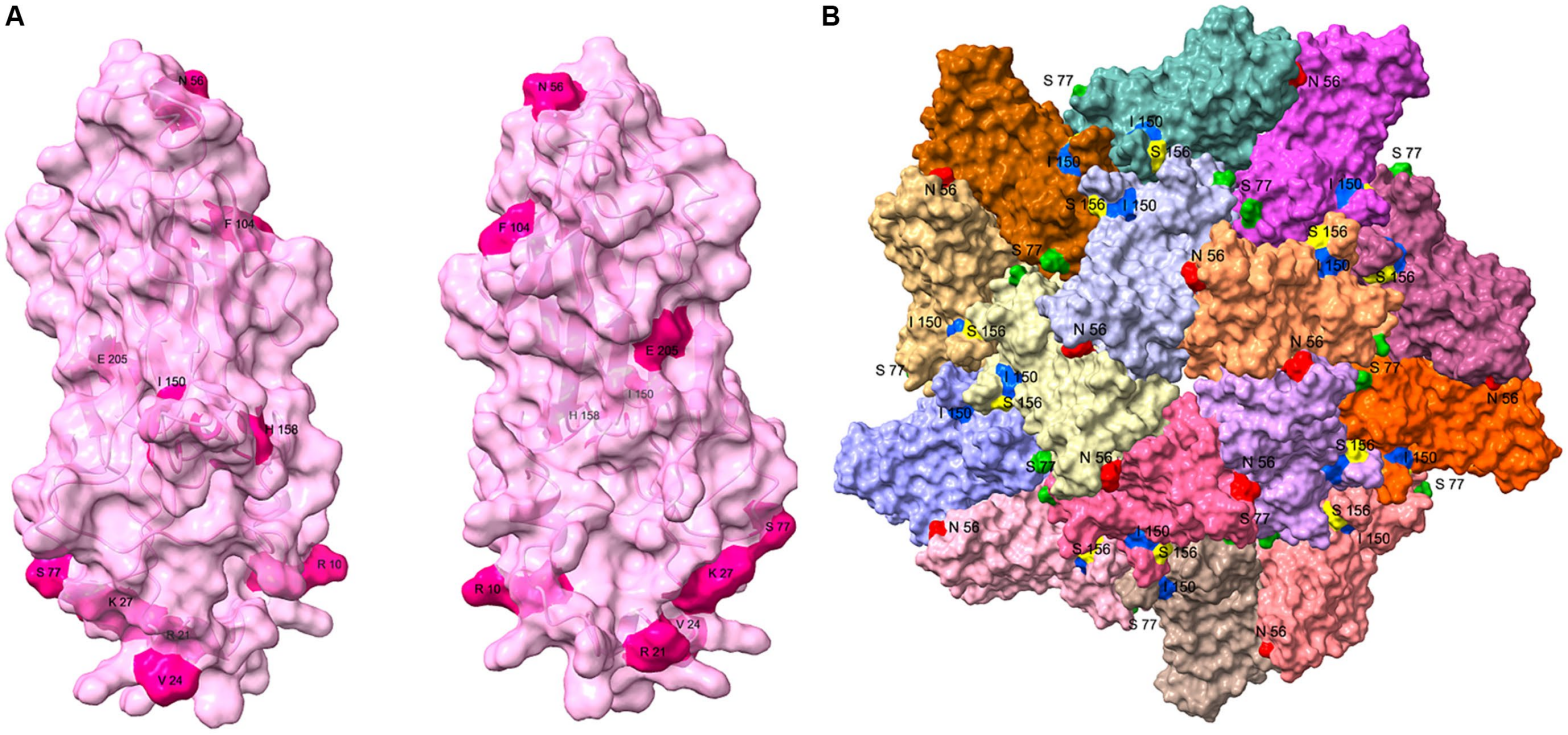
Structural analysis of PCV3 Cap protein. **(A)** Structure of the PCV3 Cap was predicted with I-TASSER, and the 10 variant sites (10, 21, 24, 27, 56, 77, 104, 150, 158, and 205) identified with the sequence alignments are presented on the structure. **(B)** Partial VLP structure of PCV3 was constructed on the PCV2 VLP template (PDB: 6OLA) with homologous modeling in SWISS MODEL. This structure contains dimers, trimmers, and pentamers of the VLP structure, and amino acids 56 (Red), 77 (Green), 150 (Blue) of the 10 variant sites, and amino acid 156 (Yellow) of the positive selection sites exposed on the surface of the VLP are shown.

The 10 variant sites identified in the sequence alignments of the PCV3 Cap were reflected in the protein structures (Figure 5A). Of the 10 variant sites, only amino acids 56, 77, and 150 were located on the surface of the VLP. Among the sites subject to positive selection pressure, only amino acid 156 was also located on the surface of the VLP (Figure 5B). In contrast, the remaining sites were not exposed on the surface of the VLP.

## 4 Discussion

PCV2 is a widespread pathogen that can infect pigs of all ages, causing PCVAD, including PMWS, PDNS, reproductive disorders, respiratory disease, and enteritis (Opriessnig et al., 2007). PCV2 is associated with subclinical infection (Opriessnig and Halbur, 2012). In addition, it may cause immunosuppression, which leads to secondary or mixed infections with other pathogens and exacerbates the clinical manifestations of PCVAD. PCV3 was first detected in 2016 in tissue samples from pigs suffering from PDNS, reproductive failure, Cardiac and multi-system inflammation in the United States. It has subsequently been detected elsewhere in the world, and currently circulating worldwide. Researchers investigated the prevalence of PCV3 in southern China during 2015–2017, and detected positive rates of 21.9, 27.8, and 31.1% in 2015, 2016, and 2017, respectively, and the positive rates varied from 7.41 to 70% across provinces (Chen et al., 2022). Other researchers tested 198 diseased materials in central China in 2018–2020, and found that the positive rate for PCV2 increased from 48.4 to 64.15% in that period and the positive rate for PCV3 increased from 36.36 to 40.96% (Xu et al., 2021). Therefore, it is evident that PCV2 and PCV3 are prevalent in our swine herds.

In this study, we established a PCV2 and PCV3 dual TaqMan qPCR and tested 1,385 samples collected from 15 provinces in China in 2020–2023. The overall positive rates for PCV2 and PCV3 were 57.95 and 37.47%, respectively, and the coinfection rate was 25.49%. PCV2 and PCV3 have high detection rates, and the possible reasons are as follows. (1) PCV2 has a high mutation rate and is evolving in an adaptive direction, so its transmissibility may be gradually increasing. (2) Although immunization with the PCV2 vaccine reduces the PCV2 viral load in swine herds, poor profits in the swine industry in the last 2 years have caused a decline in PCV2 immunization rates. (3) It is also possible that the existing vaccines are less protective against the currently prevalent strains. (4) There is no commercialized vaccine for PCV3, so its high positive rate is to be expected. Tissue samples from healthy pigs at slaughterhouses and from diseased pigs were tested and found to be 69.89 and 83.33% positive for PCV2, respectively, and 15.05 and 55.69% positive for PCV3, respectively. Although there was a difference in PCV3 positive rates between healthy and diseased pigs, there was no significant difference in the mean PCV3 viral load between them. For PCV2, the mean viral copy number was significantly higher in the diseased pigs than in the healthy pigs, demonstrating that the viral load of PCV2 correlated positively with the severity of the clinical symptoms and the pathological damage to organs. Therefore, it is not difficult to hypothesize that the ubiquitous virus PCV2 is involved in the onset and progression of diseases, to a greater or lesser extent. Testing of different organs revealed higher PCV2 loads in lymph nodes than in other organs, which was consistent with previous findings that PCV2 infection leads to lymphocyte depletion in immune organs (Segalés, 2012). However, PCV3 loads were higher in stillborn fetuses than in other organs, and the first discovery of PCV3 was in fetuses from aborted sows, suggesting that PCV3 is more closely related to reproductive disorders in sows. PCV2 directly targets immune cells, causing lymphocytopenia. Therefore, infection by other pathogens is more likely in the presence of PCV2. Fifty-six clinical samples suspected PCVAD from six provinces, co-infection of PCV2 or PCV3 with PRRSV and CSFV was highly prevalent, and a coinfection rate of the four pathogens was 19.64% (11/56). However, researchers who tested 35 samples from the three northeastern provinces found that PCV3 had a high coinfection rate with PCV2 but low coinfection rates with PRRSV and CSFV (Ha et al., 2018), inconsistent with our results. This discrepancy may be attributable to differences in the diseases present in different regions or on different pig farms.

Although PCV2 is a DNA virus, its mutation rate is similar to that of some RNA viruses. A comparison of the Cap proteins for PCV2 showed that there were specific amino acid mutation sites between two different genotypes. Among the multiple variant sites, amino acids 59, 63, 80, 86, 88, 89, 90, 91, 131, 133, 134, 137, 169, 185, 187, 191 and 206 were located in the identified epitope region (Shang et al., 2009; Mo et al., 2019). In addition, amino acids 59, 134, and 191 were the key amino acids in the broad-spectrum conformational neutralizing epitope of PCV2 (Lekcharoensuk et al., 2004; Huang et al., 2020), which leads to differences in antigenicity between genotypes. This in turn creates several immunological loopholes and facilitates the escape of PCV2 from the immune system. To reduce such possible immune loopholes, researchers have mixed different genotypes of PCV2 to prepare multivalent vaccines. Although Cap proteins from different genotypes varied greatly, with only 81.3–100% sequence identity, variations within the same genotype were often small. The homology within the PCV2a Cap protein was 91–100%, with 2–3 substitutions at the same variable site, whereas the homology between PCV2b and PCV2d was >97%, with only one amino acid difference at the same variable sites. This suggests that PCV2b and PCV2d are more stable than PCV2a, possibly due to the constant evolutionary adaptation of PCV2 to the host pig.

So far, two genotype shifts have occurred in PCV2. A shift from PCV2a to PCV2b genotypes occurred at the beginning of this century, and PCV2d gradually became the predominant genotype in 2012 (Guo et al., 2010; Xiao et al., 2015; Yang et al., 2022). Of the 64 full-length PCV2 sequences identified in this study, PCV2a accounted for 12.5%, PCV2b for 25.0%, and PCV2d for 62.5%. Why does PCV2d currently predominate? There are several possible reasons: (1) PCV2d is more likely to escape the current immunization pressures in herds; (2) PCV2d has a high transmission capacity and is more likely to spread in the herd; or (3) PCV2d may have developed a stronger resistance to the environment and disinfectants. Although there are many genotypes of PCV2 with differences in antigenicity, there is only one serotype, and cross-protection exists between genotypes has been demonstrated, but immune protection is stronger between identical genotypes than between heterozygous genotypes (Yu et al., 2023). Therefore, even if PCV2d is widespread, the available vaccines should still provide some protection to the pig population. Interestingly, sequence PCV2-JL-231-9 detected in this study belonged to the PCV2a genotype, but its Cap protein had an extra amino acid at the C-terminus, like PCV2d, whereas PCV2-HLJ-LB-2 belonged to the PCV2d genotype, but its Cap protein had no additional amino acids, as reported in previous studies (Xu et al., 2021; Yang et al., 2022). Therefore, the genotype of PCV2 cannot be directly determined simply by the amino acid profile of the C-terminal end of the Cap protein.

The Cap protein is the only structural protein of PCV. It forms the viral capsid and is the primary structural domain, which recognizes and binds to the host cell. PCV3 Cap was highly conserved, with amino acid identity ranging from 94 to 100% between strains. There were 10 variable sites, located at positions 10, 21, 24, 27, 56, 77, 104, 150, 158, and 205. Positions 24, 27, 77, 104 and 150 were the most variable sites, and the amino acids at positions 24 and 27 were considered to be molecular markers for the phylogenetic categorization of PCV3, dividing PCV3 into PCV3a and PCV3b. These two sites, together with amino acids 10 and 21, were located in the nuclear localization signal region (NLS) of the Cap protein. The NLS of PCV2 is relatively conserved, and it is yet to be investigated whether the amino acid diversity of the PCV3 NLS confers a function distinct from that of PCV2. Positions 56, 77, 150, and 156 of Cap were exposed on the surface of the VLP, and all were located in the predicted epitope region (Li et al., 2018; Jiang et al., 2020). It is hypothesized that these four amino acid sites are related to the receptor binding sites or antigenic determinants of PCV3.

There is no strict uniform standard for genotyping PCV3, and the most commonly used method is to divide it into two branches, PCV3a and PCV3b, based on whole genome sequences (Fu et al., 2018; Fux et al., 2018; Li et al., 2018). The amino acid at position 122 of the PCV3a Rep protein and the amino acids at positions 24 and 27 of the Cap protein were 122A, 24V, and 27K, respectively, whereas the corresponding amino acids of PCV3b were 122S, 24A, and 27R, respectively. PCV3a can be divided into PCV3a-1 and PCV3a-2 based on amino acids 77 and 150 of the Cap protein. Among the 18 sequences determined in this study, PCV3a-1 and PCV3b, each accounted for half of the sequences. However, the evolutionary tree based on ORF2 had four branches. Therefore, the genotyping method for PCV3 requires further improvement and nuance (Xiao et al., 2015). Based on the two criteria described above, the evolutionary tree showed that sequences from different regions of the same country and different countries were sometimes located on the same branch, indicating a high degree of homology among the PCV3 strains, with no obvious pattern in their geographic distribution.

In summary, a specific, sensitive, and reproducible dual TaqMan fluorescent qPCR for PCV2 and PCV3 was successfully established in this study, and 1,385 samples were detected with this method and sequenced. The results showed that the incidences of both PCV2 and PCV3 were high in China. The viral loads of PCV2 were significantly higher in diseased pigs than in healthy pigs, whereas the positive rate of PCV3 was higher in diseased pigs than in healthy pigs, although there was no significant difference in the viral copy numbers. PCV2d was the predominant genotype, with PCV3a and PCV3b occurring at equal levels. Both at the genomic level and at the Cap protein level, there were large differences between the various genotypes of PCV2. Compared with PCV2, PCV3 was more conserved among different strains, with no obvious pattern in its geographic distribution. These results extend our understanding of the epidemiology and variability of PCV2 and PCV3 in China, and provide a basis for the prevention and control of the infections of these two viruses.

## Data availability statement

The datasets presented in this study can be found in online repositories. The names of the repository/repositories and accession number(s) can be found at: NCBI-PP179064–PP179163.

## Ethics statement

The animal study was approved by Animal Experiments of the HVRI of CAAS. The study was conducted in accordance with the local legislation and institutional requirements.

## Author contributions

MC: Writing – original draft, Software, Validation, Data curation. YW: Writing – review & editing, Resources, Supervision. WS: Writing – review & editing, Resources, Supervision. LF: Writing – review & editing, Funding acquisition, Resources, Supervision. LH: Writing – review & editing, Data curation, Funding acquisition, Resources, Supervision.

## Footnotes

^1^https://www.chiplot.online/tvbot.html.

^2^http://www.datamonkey.org

^3^http://zhanglab.dcmb.med.umich.edu

^4^https://swissmodel.expasy.org
