# Supplementary material for: Investigation of porcine circovirus type 2 and porcine circovirus type 3 infections based on dual TaqMan fluorescent quantitative PCR method and genetic evolutionary analysis of these two viruses
Source: Front Microbiol. 2024 Mar 14;15:1385137. doi: 10.3389/fmicb.2024.1385137 (PMC10972944; doi:10.3389/fmicb.2024.1385137)
Supplement: Supplementary file 1 [file Table_1.DOCX]

Supplementary Material

# Supplementary Tables

**Table S1. Information about PCV2 and PCV3 isolates obtained in this study**

| No. | GenBank accession no. | | Strain name | | Geographic location | Collection date | | | Length(bp) |
| --- | --- | --- | --- | --- | --- | --- | --- | --- | --- |
| 1 | PP179082 | PCV2-JL-LB-6 | | China:Jilin | | | 2023 | 1767 | |
| 2 | PP179083 | PCV2-JL-LB-13 | | China:Jilin | | | 2023 | 1768 | |
| 3 | PP179084 | PCV2-JL-LB-30 | | China:Jilin | | | 2023 | 1767 | |
| 4 | PP179085 | PCV2-JL-LB-79 | | China:Jilin | | | 2023 | 1766 | |
| 5 | PP179086 | PCV2-JL-LB-163 | | China:Jilin | | | 2023 | 1767 | |
| 6 | PP179087 | PCV2-JL-LB-238 | | China:Jilin | | | 2023 | 1766 | |
| 7 | PP179088 | PCV2-JL-LB-282 | | China:Jilin | | | 2023 | 1767 | |
| 8 | PP179089 | PCV2-JL-425 | | China:Jilin | | | 2023 | 1767 | |
| 9 | PP179090 | PCV2-JL-519 | | China:Jilin | | | 2023 | 1767 | |
| 10 | PP179091 | PCV2-JL-512 | | China:Jilin | | | 2023 | 1767 | |
| 11 | PP179092 | PCV2-JL-554 | | China:Jilin | | | 2023 | 1767 | |
| 12 | PP179093 | PCV2-JL-557 | | China:Jilin | | | 2023 | 1767 | |
| 13 | PP179094 | PCV2-JL-606 | | China:Jilin | | | 2023 | 1767 | |
| 14 | PP179095 | PCV2-JL-685 | | China:Jilin | | | 2023 | 1767 | |
| 15 | PP179096 | PCV2-JL-687 | | China:Jilin | | | 2023 | 1768 | |
| 16 | PP179097 | PCV2-HeN-15 | | China:Heinan | | | 2023 | 1767 | |
| 17 | PP179098 | PCV2-XJ-21 | | China:Xinjiang | | | 2023 | 1767 | |
| 18 | PP179099 | PCV2-GX-35 | | China:Guangxi | | | 2023 | 1767 | |
| 18 | PP179100 | PCV2-HN-61 | | China:Hunan | | | 2023 | 1767 | |
| 20 | PP179101 | PCV2-GD-30 | | China:Guangdong | | | 2023 | 1767 | |
| 21 | PP179102 | PCV2-GX-27 | | China:Guangxi | | | 2023 | 1767 | |
| 22 | PP179103 | PCV2-SX-433 | | China:Shanxi | | | 2023 | 1767 | |
| 23 | PP179104 | PCV2-SX-430 | | China:Shanxi | | | 2023 | 1767 | |
| 24 | PP179105 | PCV2-SX-4007 | | China:Shanxi | | | 2023 | 1767 | |
| 25 | PP179106 | PCV2-JL-324 | | China:Jilin | | | 2023 | 1767 | |
| 26 | PP179107 | PCV2-HLJ-64 | | China:Heilongjiang | | | 2023 | 1767 | |
| 27 | PP179108 | PCV2-HLJ-R2 | | China:Heilongjiang | | | 2023 | 1767 | |
| 28 | PP179109 | PCV2-JS-26-2 | | China:Jiangsu | | | 2023 | 1767 | |
| 29 | PP179110 | PCV2-HLJ-W2 | | China:Heilongjiang | | | 2023 | 1767 | |
| 30 | PP179111 | PCV2-LN-13 | | China:Liaoning | | | 2023 | 1767 | |
| 31 | PP179112 | PCV2-HeB-15 | | China:Heibei | | | 2023 | 1767 | |
| 32 | PP179113 | PCV2-HLJ-19 | | China:Heilongjiang | | | 2023 | 1767 | |
| 33 | PP179114 | PCV2-HeB-21-2 | | China:Heibei | | | 2023 | 1767 | |
| 34 | PP179115 | PCV2-HeB-24-2 | | China:Heibei | | | 2023 | 1767 | |
| 35 | PP179116 | PCV2-JS-25-2 | | China:Jiangsu | | | 2023 | 1767 | |
| 36 | PP179117 | PCV2-NMG-35 | | China:Neimenggu | | | 2023 | 1767 | |
| 37 | PP179118 | PCV2-LN-46 | | China:Liaoning | | | 2023 | 1767 | |
| 38 | PP179119 | PCV2-YN-1 | | China:Yunnan | | | 2023 | 1767 | |
| 39 | PP179120 | PCV2-HeB-22-2 | | China:Heibei | | | 2023 | 1767 | |
| 40 | PP179121 | PCV2-SD-4 | | China:Shandong | | | 2023 | 1767 | |
| 41 | PP179122 | PCV2-SD-44 | | China:Shandong | | | 2023 | 1767 | |
| 42 | PP179123 | PCV2-LN-9 | | China:Liaoning | | | 2023 | 1767 | |
| 43 | PP179124 | PCV2-JL-14-16 | | China:Jilin | | | 2023 | 1767 | |
| 44 | PP179125 | PCV2-JX-18 | | China:Jiangxi | | | 2023 | 1767 | |
| 45 | PP179126 | PCV2-LN-21 | | China:Liaoning | | | 2023 | 1767 | |
| 46 | PP179127 | PCV2-HLJ-15-3 | | China:Heilongjiang | | | 2023 | 1767 | |
| 47 | PP179128 | PCV2-HLJ-LB-2 | | China:Heilongjiang | | | 2023 | 1767 | |
| 48 | PP179129 | PCV2-JL-231-9 | | China:Jilin | | | 2023 | 1767 | |
| 49 | PP179130 | PCV2-NMG-1-8 | | China:Neimenggu | | | 2023 | 1768 | |
| 50 | PP179131 | PCV2-NMG-1-4 | | China:Neimenggu | | | 2023 | 1767 | |
| 51 | PP179132 | PCV2-NMG-16-9 | | China:Neimenggu | | | 2023 | 1767 | |
| 52 | PP179133 | PCV2-HLJ-44-2 | | China:Heilongjiang | | | 2023 | 1768 | |
| 53 | PP179134 | PCV2-HLJ-36-1 | | China:Heilongjiang | | | 2023 | 1768 | |
| 54 | PP179135 | PCV2-HLJ-22-7 | | China:Heilongjiang | | | 2023 | 1767 | |
| 55 | PP179136 | PCV2-JL-23-8 | | China:Jilin | | | 2023 | 1767 | |
| 56 | PP179137 | PCV2-LN-24-2 | | China:Liaoning | | | 2023 | 1767 | |
| 57 | PP179138 | PCV2-LN-47-5 | | China:Liaoning | | | 2023 | 1767 | |
| 58 | PP179139 | PCV2-HLJ-462-3 | | China:Heilongjiang | | | 2023 | 1767 | |
| 59 | PP179140 | PCV2-HLJ-491-1 | | China:Heilongjiang | | | 2023 | 1767 | |
| 60 | PP179141 | PCV2-NMG-57-13 | | China:Neimenggu | | | 2023 | 1767 | |
| 61 | PP179142 | PCV2-JX-27-12 | | China:Jiangxi | | | 2023 | 1768 | |
| 62 | PP179143 | PCV2-NMG-5-13 | | China:Neimenggu | | | 2023 | 1766 | |
| 63 | PP179144 | PCV2-NMG-5-14 | | China:Neimenggu | | | 2023 | 1768 | |
| 64 | PP179145 | PCV2-LN-14-5-1 | | China:Liaoning | | | 2023 | 1767 | |
| 65 | PP179064 | PCV3-HLJ-R2 | | China:Heilongjiang | | | 2023 | 2000 | |
| 66 | PP179065 | PCV3-NMG-B22-1 | | China:Neimenggu | | | 2023 | 2000 | |
| 67 | PP179066 | PCV3-YN-B2 | | China:Yunnan | | | 2023 | 2000 | |
| 68 | PP179067 | PCV3-HLJ-XD-62 | | China:Heilongjiang | | | 2023 | 2000 | |
| 69 | PP179068 | PCV3-SXXA-XD-410 | | China:Shanxi,Xian | | | 2023 | 2000 | |
| 70 | PP179069 | PCV3-MDJ-XD-376 | | China: Heilongjiang | | | 2023 | 2000 | |
| 71 | PP179070 | PCV3-MDJ-XD-363 | | China: Heilongjiang | | | 2023 | 2000 | |
| 72 | PP179071 | PCV3-LN-14-5-4 | | China:Liaoning | | | 2023 | 2000 | |
| 73 | PP179072 | PCV3-JL-231 | | China:Jilin | | | 2023 | 2000 | |
| 74 | PP179073 | PCV3-NMG-53-3 | | China:Neimenggu | | | 2023 | 2000 | |
| 75 | PP179074 | PCV3-NMG-55-6 | | China:Neimenggu | | | 2023 | 2000 | |
| 76 | PP179075 | PCV3-NMG-C1-2 | | China:Neimenggu | | | 2023 | 2000 | |
| 77 | PP179076 | PCV3-NMG-C6-4 | | China:Neimenggu | | | 2023 | 2000 | |
| 78 | PP179077 | PCV3-NMG-C9-4 | | China:Neimenggu | | | 2023 | 2000 | |
| 79 | PP179078 | PCV3-JL-14-21-4 | | China:Jilin | | | 2023 | 2000 | |
| 80 | PP179079 | PCV3-NMG-D5-3 | | China:Neimenggu | | | 2023 | 2000 | |
| 81 | PP179080 | PCV3-NMG-12-1 | | China:Neimenggu | | | 2023 | 2000 | |
| 82 | PP179081 | PCV3-NMG-57-7 | | China:Neimenggu | | | 2023 | 2000 | |
| 83 | PP179146 | PCV3-NMG-B22-1 | | China:Neimenggu | | | 2023 | 645 | |
| 84 | PP179147 | PCV3-YN-B2 | | China:Yunnan | | | 2023 | 645 | |
| 85 | PP179148 | PCV3-NMG-B43 | | China:Neimenggu | | | 2023 | 645 | |
| 86 | PP179149 | PCV3-JL-B27 | | China:Jilin | | | 2023 | 645 | |
| 87 | PP179150 | PCV3-LN-B12 | | China:Liaoning | | | 2023 | 645 | |
| 88 | PP179151 | PCV3-HLJ-W2 | | China:Heilongjiang | | | 2023 | 645 | |
| 89 | PP179152 | PCV3-SXXA-446 | | China:Shanxi,Xian | | | 2023 | 645 | |
| 90 | PP179153 | PCV3-MDJ-363 | | China: Heilongjiang | | | 2023 | 645 | |
| 91 | PP179154 | PCV3-JL-277 | | China:Jilin | | | 2023 | 645 | |
| 92 | PP179155 | PCV3-JL-14-5-4 | | China:Jilin | | | 2023 | 645 | |
| 93 | PP179156 | PCV3-JL-231 | | China:Jilin | | | 2023 | 645 | |
| 94 | PP179157 | PCV3-NMG-53-3 | | China:Neimenggu | | | 2023 | 645 | |
| 95 | PP179158 | PCV3-SD-13-2 | | China:Shandong | | | 2023 | 645 | |
| 96 | PP179159 | PCV3-LN-14-21-4 | | China:Liaoning | | | 2023 | 645 | |
| 97 | PP179160 | PCV3-NMG-D5-3 | | China:Neimenggu | | | 2023 | 645 | |
| 98 | PP179161 | PCV3-JX-25 | | China:Jiangxi | | | 2023 | 645 | |
| 99 | PP179162 | PCV3-HLJ-7 | | China:Heilongjiang | | | 2023 | 645 | |
| 100 | PP179163 | PCV3-NMG-6 | | China:Neimenggu | | | 2023 | 645 | |
